# Visual categories and concepts in the avian brain

**DOI:** 10.1007/s10071-022-01711-8

**Published:** 2022-11-10

**Authors:** Roland Pusch, William Clark, Jonas Rose, Onur Güntürkün

**Affiliations:** 1 Biopsychology, Faculty of Psychology, Ruhr University Bochum, 44780 Bochum, Germany; 2 Neural Basis of Learning, Faculty of Psychology, Ruhr University Bochum, 44780 Bochum, Germany

**Keywords:** Pigeon, Tectofugal, Sensory cortex, Common elements theory, Brain asymmetry

## Abstract

Birds are excellent model organisms to study perceptual categorization and concept formation. The renewed focus on avian neuroscience has sparked an explosion of new data in the field. At the same time, our understanding of sensory and particularly visual structures in the avian brain has shifted fundamentally. These recent discoveries have revealed how categorization is mediated in the avian brain and has generated a theoretical framework that goes beyond the realm of birds. We review the contribution of avian categorization research—at the methodical, behavioral, and neurobiological levels. To this end, we first introduce avian categorization from a behavioral perspective and the common elements model of categorization. Second, we describe the functional and structural organization of the avian visual system, followed by an overview of recent anatomical discoveries and the new perspective on the avian ‘visual cortex’. Third, we focus on the neurocomputational basis of perceptual categorization in the bird’s visual system. Fourth, an overview of the avian prefrontal cortex and the prefrontal contribution to perceptual categorization is provided. The fifth section outlines how asymmetries of the visual system contribute to categorization. Finally, we present a mechanistic view of the neural principles of avian visual categorization and its putative extension to concept learning.

“But, unless there is something extraordinary about the conceptual capacities of pigeons, our findings show that an animal readily forms a broad and complex concept when placed in a situation that demands one”. Herrnstein and Loveland (1964, p. 551)

## Birds master a sheer endless variety of perceptual categories

The critical function of any brain is to predict the consequences of actions based on sensory stimuli. Analysis of sensory input can be rather simple, for instance when consuming a standardized food item that is directly in the field of view. But often decisions involve a wealth of past experiences and a complex sensory analysis since not all stimuli that require the same action also look the same. Perceptual categorization enables animals to group stimuli based on their sensory features (see Box 1 for formal definitions). This core cognitive ability is executed almost instantaneously, seemingly without any effort, and allows assigning functional associations to items in the world around us. In fact, *categorization* appears at a comparable timescale as the initial *detection* of an object. The category membership can be reported before an idiosyncratic *identification* of an object is possible (Grill-Spector and Kanwisher 2005). As a result of these operations, organisms handle the endless variety of perceptual input by first *recognizing* the category of items to subsequently *discriminate* between them or *generalize* across different stimuli. All these different processes contribute to *categorization* and the formation of *concepts*. How categorization is mediated at a neuronal level, what stimulus features are used, and how concepts emerge from categories remain open questions. These mechanisms have previously been reviewed (Soto and Wasserman 2014) and synthesized into a mechanistic hypothesis (Güntürkün et al. 2018). In the current review, we will provide insights from the realm of birds into the behavior and the neurobiology of perceptual visual categorization by mainly focusing on key developments of recent years. Although we only review studies that used visual stimuli, there is strong evidence from experiments using human participants that categorization of visual and tactile objects generates highly similar veridical perceptual spaces to form overlapping object categorization processes (Tabrik et al. 2021). Studies in corvids also show that auditory categorization follows highly similar principles to the visual system (Wagener and Nieder 2020).

Box 1 Before we can delve into the details of perceptual categorization, some initial definitions should be given to unambiguously separate distinct processes. This is especially important since the terms are sometimes used with slightly different connotations. These definitions are in part reviewed in Huber and Aust (2017), Lazareva and Wasserman (2017), and Palmeri and Gauthier (2004):

**Detection** refers to the ability to report the mere existence of an object in a visual scene. This is the basic behavioral readout in visual processing. Beyond detecting a visual object are the processes of object recognition and identification.

**Recognition** is the ability of determining that one has already seen an object before no matter if one knows what it is, i.e., also without being able to categorize it. A special case of object recognition is its subsequent **Identification.** The term refers to an object’s unique identity rather than to a member of a category. Detection, recognition and identification relate to single objects. These processes do not consider the relationship between different objects.

**Discrimination** refers to the ability to distinguish between two or more stimuli. This ability is thought to be based on detected differences and despite perceived similarities among stimuli. An animal can be said to discriminate when it has learned to respond differently to two or more stimuli. Discrimination is the process by which members of the same category can be distinguished and is based on unique stimulus features albeit shared stimulus features exist.

Opposed to the discrimination of objects is a process termed **Generalization**. It is the ability to group two or more stimuli on the basis of detected similarities and despite perceived differences among them. An animal can be said to generalize when it has learned to respond in the same or similar way to two or more different stimuli. Generalization is the process by which members of the same category are bound together, often by extrapolation along a physical dimension.

**Categorization** entails the process of determining which objects belong together and share a common class-membership. Thus, to categorize means to distinguish between in-category and out-category instances based on perceptual similarity. A category is a quantity of stimuli that all share common stimulus features. The process of categorization is open-ended and can importantly be applied to new instances (Herrnstein 1990). Categorization can be realized at different levels of abstraction (Huber and Aust 2017; Lazareva et al. 2004). If stimuli form a **perceptual category** they are grouped based on features, i.e., perceivable physical similarity of the stimulus material. This grouping will be easier, if there is large physical similarity between the stimuli of one category and sufficient physical difference to non-category members, often reflected in basic-level categories. If similarity increases and instances have to be excluded despite shared perceptual features, categorization takes place at the subordinate level. The inclusion of more and more basic-level categories leads to a gradual decrease in physical similarity, reflecting categorization at the superordinate level. However, to categorize stimuli either on the sub- or superordinate level requires intensive learning compared to basic-level categorization.

If stimuli are grouped together albeit not all instances share the same perceptual features, **concepts** emerge. On a higher cognitive level, stimuli can be bound together as a concept when they signal identical consequences. These stimuli build so-called equivalence classes. The process of forming a concept can also be based on similar functions or actions required. Importantly, the formation of concepts is not based on perceptual similarity across all instances. On the most abstract level, concepts are based on the relations between instances (relations: e.g., same vs. different or transitive inference).

Categorization is a widespread cognitive capacity across the animal kingdom and, therefore, must have high adaptive significance (Lazareva and Wasserman 2017). The seminal study by Herrnstein and Loveland (1964) marked the starting point for the comparative research on categorization by showing that pigeons learn the category “human” after being conditioned to discern a large number of photographs of which some depicted humans while others did not. The main point that the study revealed was that the birds not only discriminated between the training stimuli but subsequently also transferred their knowledge to photographs that they had never seen before. The animals were able to generalize their knowledge to new instances indicative of an open-ended category beyond rote memorization (Herrnstein 1990). This generalization test is the hallmark of categorization learning. For categorization to be successful, animals have to memorize the common features of a category, and at that level, categorization learning and rote memory overlap. However, during rote memorization, stimulus-unique features are also learned which can only be used to remember a singular object. Taken together, during a perceptual category learning task as used by Herrnstein and Loveland (1964), stimuli are grouped based on the perceived physical similarity of their common features. This grouping is learned faster, the more the stimuli of one category resemble each other and the more dissimilar they are to non-category members (see Box 1 for formal definitions).

Numerous behavioral and recently neurophysiological studies in birds broadened our understanding of the mechanisms guiding categorization behavior. Since pigeons are among the most thoroughly studied non-human species with respect to categorization learning, a comprehensive body of literature has emerged (for recent reviews, see: Güntürkün et al. 2018; Huber and Aust 2017; Soto and Wasserman 2014). Here, we list some spectacular cases: categorization abilities of pigeons have been employed by the American armed forces in experimental studies to recognize ships and guide missiles towards them (Skinner 1960), more peaceful minded pigeons learned to categorize the orthography of four-letter words (Scarf et al. 2016), to categorize paintings as cubist or impressionist (Anderson et al. 2020; Watanabe et al. 1995), to distinguish between aerial images showing man-made and natural structures (Lubow 1974), and to properly distinguish benign from malignant human breast histopathological images (Levenson et al. 2015). These examples highlight that the ability to categorize is almost independent of the perceptual content of the actual stimulus classes. A multitude of different protocols enable the testing and fine-grained investigation of what dimensions of stimulus features drive categorization behavior.

Most of the initial investigations of categorization behavior have been performed with Go/NoGo-tasks. For example, in the above-mentioned study by Herrnstein and Loveland (1964), pigeons were rewarded when pecking on a picture containing humans (Go stimulus). A pecking response on pictures not containing humans was not rewarded (NoGo stimulus). Other approaches often used in categorization research are forced-choice procedures. In its easiest form, a two-alternative forced-choice procedure, many dichotomous categorization tasks have been performed in a variety of species (e.g., Roberts and Mazmanian 1988). In these tasks, two stimuli are presented simultaneously, and the animals base their choice on the category membership of the stimuli. Throughout the history of categorization research, many paradigms that were more sophisticated have been employed to further investigate the categorization abilities of pigeons. In a four-alternative choice procedure, Bhatt et al. (1988) could show that pigeons are able to learn four different categories simultaneously (cats, flowers, cars, and chairs). No difference in learning speed with respect to the nature of the different stimuli could be detected. The authors further showed that repetitive stimulus presentation is not mandatory for category learning, albeit it aids the categorization process and increases the learning speed. Using the same four categories, Lazareva et al. (2004) could show that pigeons categorize stimuli based on different hierarchical levels. In a basic-level approach, the animals learned to distinguish stimuli of “cars”, “chairs”, “flower” and “people” in a four-alternative choice procedure. Concurrently, the task involved stimulus groupings on the superordinate level. Here, the stimuli of the categories “flower” and “people” were grouped together as a combined superordinate concept “natural”. This concept was then distinguished from the combined superordinate concept “artificial” composed of the stimuli of the basic-level categories “chairs” and “cars”. It turned out that pigeons were successful in learning both levels of categories at the same time, although they were faster in acquiring those at the basic level (Lazareva et al. 2004). Most importantly, the superordinate concept “natural” was learned rather quickly, possibly because the stimuli of the two natural categories resembled each other more than those of the two artificial categories. This possibly represents a nice example for “semantic” categories that are often constituted by rather distant members that still have some overlapping perceptual features.

In a series of following experiments, the categorization abilities were investigated in up to 16-alternative forced-choice procedures (Wasserman et al. 2015). In these experiments, birds were found to be able to learn a vast variety of stimulus categories in parallel. In pseudocategorization experiments employing random stimulus assignments, the birds were still able to group these diverse stimuli, though learning was far slower than when category membership is defined by perceptual coherence (Herrnstein and Villiers 1980; Wasserman et al. 1988). Thus, categorization is an important mental shortcut to group vast amounts of stimuli that are hard to learn by rote memory. When 3-D objects in different viewpoints were used as stimuli, pigeons showed a strong and instantaneous tendency to categorize shapes of geometric objects that were presented in different rotations. In this study, the animals were unable to learn a pseudocategorization, possibly due to the fact that the randomly chosen borders of pseudocategories contradicted the highly visible natural category borders (Peissig et al. 2019).

It is likely that pigeons also learn abstract concepts that have no clear perceptual overlapping structure. An example is the dichotomy same vs. different. Here, the concept depends on the relation between stimulus pairs. Since these pairs might not have any perceptual feature in common, the birds have to solve the task by a rule. Once this rule is learned, any trial can be correctly solved. Wright et al. (1988) showed that pigeons that worked daily on 152 stimulus pairs from a set of 232 pictures performed a successful transfer to novel instances. In contrast, birds trained on a much smaller training set did not. Therefore, pigeons are able to learn an abstract concept, but they need lengthy training procedures and may require many examples to do so properly. Crows, in contrast, learn abstract rules and concepts much faster (Veit and Nieder 2013; Nieder 2021). Several further examples of concept learning in birds are discussed in Lazareva and Wasserman (2017). We will discuss the possible reasons for species-specific limitations of concept learning below.

In a nutshell, birds can group a vast variety of visual stimuli into categories and concepts at different levels of abstraction. Thus, a continuum spans from idiosyncratic stimulus identification to category assignment at the superordinate level to abstract concepts that integrate more complex features of the visual input. However, the variety of different tasks, the diverse stimulus sets used and the different training schemes employed stresses the question about a unifying mechanism underlying these impressive capabilities (Huber and Aust 2017). The general process of perceptual categorization and the formation of concepts seems to rely on the extraction of visual stimulus features, to sort these features into categories and concepts at different levels of abstraction and to opt for a behavioral response with respect to the task demand at hand. However, what features do the animals select when categorizing different stimuli?

## The common elements model of categorization

The common elements model of categorization (Soto and Wasserman 2010, 2012) provides a theoretical and neurobiological framework that describes how the avian visual system parcellates objects into different categories and uses these representations to guide decision making. The model rests on two assumptions. First, objects belonging to a category are represented by a combination of shared perceptual features (the elements), and these elements have different probabilities of being a diagnostic measure of a particular category. Elements that have high probability of diagnosing a particular category are shared between many, if not all different objects, making these elements category-specific. In contrast, elements that have a low probability of diagnosing a particular category are not shared by many objects comprising the category, making these elements only stimulus-specific. Second, the model assumes that connections between category-specific or stimulus-specific elements and behavioral responses are strengthened through error-driven learning, depending on their ability to predict reward. As learning is proportionate to reward-prediction error, only stimulus-specific and category-specific elements that are predictive of reward control behavioral decisions.

The common elements model is implemented as a simple hierarchical feedforward network (Riesenhuber and Poggio 2000; Serre et al. 2007), with alternating simple cell-like and complex cell-like layers as inspired by the architecture of the mammalian ventral visual stream. This pathway is a recurrent occipito-temporal network that associates early visual areas with the anterior inferior temporal cortex, and shows diverse and clustered categorical selectivity for visual objects (Kravitz et al. 2013). Thereby, layers of simple cells are interleaved with layers of complex cells, which combine the input of several units with similar selectivity but slightly different positions and scales. These non-linear operations between layers allow the network to extract increasingly specific and complex image features, mimicking the hierarchical computations known to occur along the pigeon tectofugal pathway (Li et al. 2007; Azizi et al. 2019). Some aspects of the model are not completely consistent with the physiology of the primate visual system. For instance, final layer neurons do not show invariance and sparseness comparable with inferior temporal cortex (Robinson and Rolls 2015). The model is, however, a reasonable approximation of the simple hierarchical processing operations that occur along the tectofugal pathway in the avian brain.

For classification learning, complex units across the four layers of complex cells of the common elements model project directly to a reinforcement learning stage. The reinforcement learning stage replicates the function of the dopaminergic system, which computes reward-prediction error in conjunction with the prefrontal cortex (PFC) in mammals (Starkweather et al. 2018) and the functionally analogous structure, nidopallium caudolaterale (NCL) in birds (Packheiser et al. 2021). These operations are mediated by dopamine projections, which is a key stage enabling the organism to select the appropriate category signal emanating from the PFC/NCL to make an appropriate motor response (Antzoulatos and Miller 2011; Puig and Miller 2012; Schultz 2016). Reward-driven feedback also allows PFC/NCL to shape the responses of neurons in the visual cortex (Sasikumar et al. 2018). Soto and Wasserman (2012) revealed that the common elements model captures most of pigeons’ behavioral performance in categorization tasks (e.g., size transformation, view interpolation, and surface feature removal). Interestingly, the model more closely approximated pigeon than human behavior in several of the experimental designs tested, aligning with the evidence that pigeons show substantially less capacity to tolerate transformations across viewpoint, size, location etc. (Soto and Wasserman 2014). These findings suggest that some components of the primate visuo-spatial system, PFC and extended memory systems that enable higher-order object recognition abilities (e.g., “mental rotation” and view interpolation) do not have equivalents in the pigeon brain. As we will discuss when we turn our attention to the avian visual cortex, these findings align with the neurophysiological data suggesting that the pigeon visual system represents object features at an intermediate stage of complexity relative to primates (Clark and Colombo 2022; Clark et al. 2022a). We here use the term “avian visual cortex” to label the isocortex-like pallial components of the visual thalamofugal and tectofugal pathways (Stacho et al. 2020). This will be outlined below.

## A short overview of the avian visual pathways

**Fig. 1:**
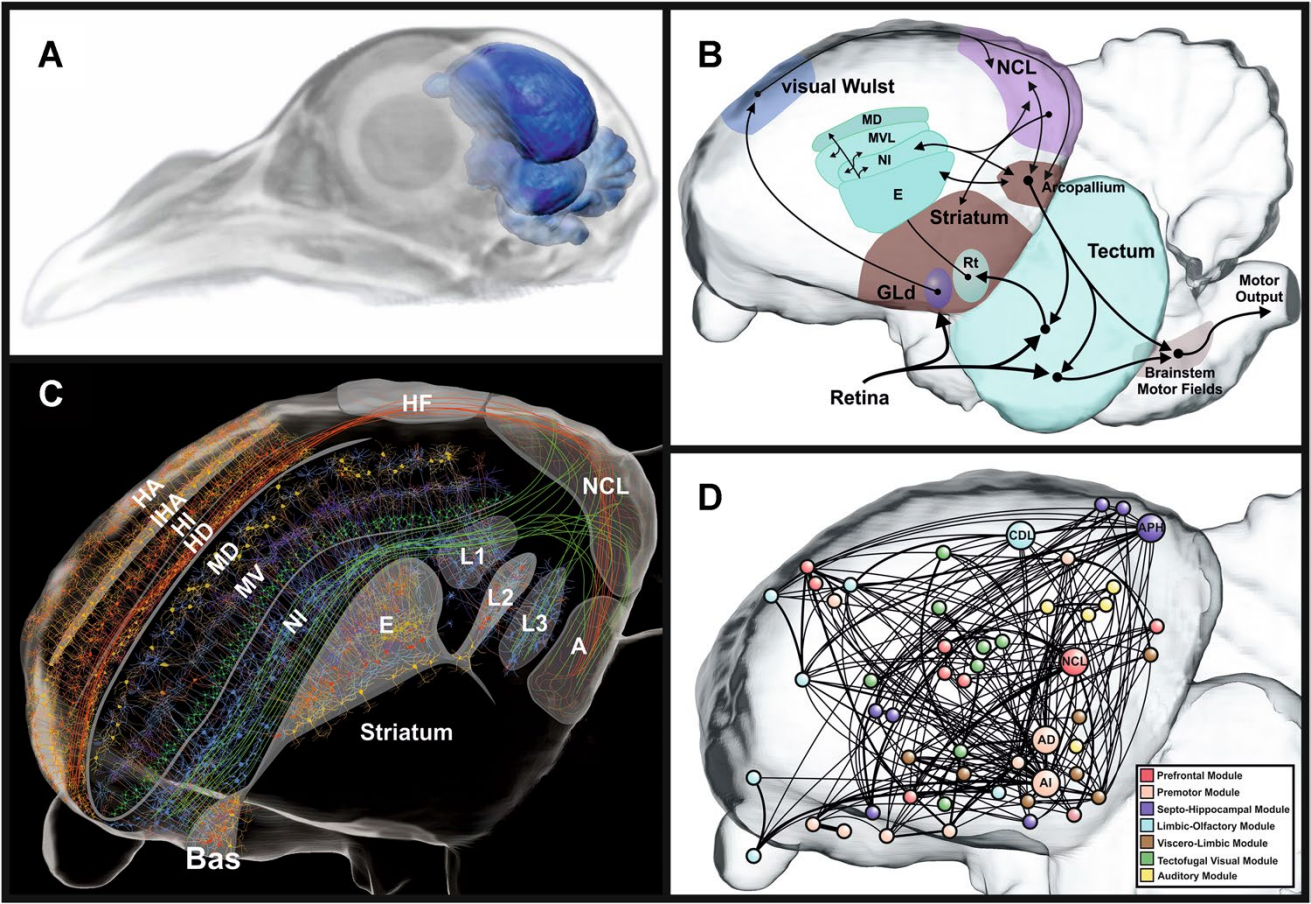
Schematic anatomical arrangement of the pigeon brain, the visual pathways and the forebrain connectome. **A** Overlap of MRI pigeon brain within the CT head data and the pigeon’s brain position. As transparent structures, the eye and the embedding skull are visible. Please note the large eye and the enlarged cerebrum. **B** Sagittal depiction of the visual pathways in the pigeon brain. Birds have two main visual pathways known as the tectofugal and thalamofugal systems that correspond to the mammalian extrageniculocortical and geniculocortical pathways, respectively. **C** Sagittal view of the pigeon forebrain with a highly schematized depiction of the avian sensory cortex. Adapted from Güntürkün et al. (2021). **D** The connectome of the pigeon cerebrum in sagittal view. Nodes are color-coded according to module membership. Based on the study by Shanahan et al. (2013). *A* arcopallium; *AD*: arcopallium dorsale, *AI* arcopallium intermedium, *APH* area parahippocampalis, *Bas* N. basalis prosencephalic, *CDL* area corticoidea dorsolateralis, *E* entopallium, *GLd* nucleus geniculatus lateralis, pars dorsalis, *HA* hyperpallium apicale, *HD* hyperpallium dorsale, *HF* hippocampal formation, *HI* hyperpallium intercalatum, *IHA* N. interstitialis hyperpallii apicalis, *L1-3* field L1-3, *MD* mesopallium dorsale, *MV* mesopallium ventral, *NCL* Nidopallium caudolaterale, *NI* Nidopallium intermedium, *Rt* nucleus rotundus

In 1943, the French ophthalmologist André Rochon-Duvigneaud coined pigeons as nothing else but two eyes with wings. We humans are highly visual primates and view our surroundings with the information transmitted by about 1 million axons within each of our optic nerves. In pigeons with their 2.5 g brain, this number stands at 2.3 million (Binggeli and Paule 1969). Pigeons also surpass humans in their ability to discriminate luminance (Hodos et al. 1985), and discern subtle color differences (Emmerton and Delius 1980). Birds have exceptionally large eyes for their body size and their cerebrum is enlarged by at least a factor of 10 relative to similarly sized fish and reptiles (Shimizu et al. 2017). Figure 1A exemplifies these findings for pigeons. To facilitate the fine-grained analysis of objects features, the avian retina is equipped with two specialized regions of high cone and ganglion cell densities to enhance spatial and temporal resolution (Bringmann et al. 2018). These two areas have different projections to neural structures and enable distinct analyses of the visual input (Remy and Güntürkün 1991; Güntürkün and Hahmann 1999; Clark and Colombo 2022). The avian tectofugal pathway—homologous to the mammalian extrageniculocortical pathway—is mainly responsible for both object and motion vision in the frontal visual field: As depicted in Fig. 1B, visual information travels from the eye to the midbrain optic tectum and thence to the nucleus rotundus in the thalamus. From here, the information flow enters the entopallium, one of the two primary visual areas of the telencephalon, and is further relayed to multiple higher visual associative forebrain areas. The thalamofugal pathway is homologous to the mammalian geniculocortical pathway and processes visual information from the lateral field of view. The visual information from the retina travels via the nucleus geniculatus lateralis, pars dorsalis (GLd) in the thalamus to the visual Wulst in the telencephalon (Clark and Colombo 2020). These visual pathways divide and process information in a spatially parallel manner (Nguyen et al. 2004), utilizing a cellular architecture constituted by columnar local connections and horizontal layers in hyperpallium and DVR, that resembles the mammalian cortex in terms of its anatomical (Fig. 1C; Stacho et al. 2020) and its network structure (Fig. 1D).

Among other vertebrates, only mammals display a comparably enlarged cerebrum like birds, with primates possessing exceptionally high neuron densities like corvids and parrots (Kverková et al. 2022). Many mammals (particularly primates) also possess large eyes for their body size (Ross and Kirk 2007), developed fovea (Provis et al. 1998), and greatly expanded visual processing networks in the telencephalon (Kaas et al. 2022; van Essen et al. 1992). The similarities between birds and primates means that understanding the physiology of the avian visual system represents a unique opportunity to compare how similar principles of perception, motor control and planning are implemented by neuronal hardware that differs from the mammalian cortex.

## The thalamofugal pathway

The study of Stacho et al. (2020) demonstrated that the entire sensory pallium of birds encompassing both the components of the visual thalamofugal and the visual tectofugal systems is characterized by columnar canonical iterative circuits that are highly similar in both the thalamofugal and the tectofugal regions. Thus, these circuits are mostly identical throughout sensory systems and pallial areas (canonical) and they are repeated in identical way throughout the expanse of the sensory pallium. In addition, canonical circuits of both thalamo- and tectofugal systems are tangentially intersected by long-range associative axons that cross-connect all columns and link them to prefrontal, hippocampal, and (pre)motor structures (Fig. 1C). This cortical organization is only visible in the sensory pallium, while associative and motor areas have a different organization. The thalamofugal visual system terminates in the cortex-like territory of the Wulst (German for bulge or swelling), a laminated structure at the dorsal roof of the avian telencephalon which contains both a somatosensory and a visual processing region (Bischof et al. 2016; Pettigrew and Konishi 1976a, b; Wild 1987). This visual component of the Wulst receives projections from the dorsolateral geniculate nucleus (GLd) and constitutes together with the GLd the thalamofugal visual pathway (Güntürkün and Karten 1991). The Wulst is functionally analogous with the primary visual cortex (V1) in many respects, such as displaying detailed retinotopic maps of the visual space, selectivity to orientation/direction of motion, and small receptive field sizes (Bischof et al. 2016; Gusel'nikov et al. 1977; Revzin 1969). In predatory birds with frontally oriented eyes, such as owls, the cortex-like architecture of the Wulst is expanded which may be related to their behavioral specializations. In these birds, the Wulst plays an important role in computing binocular disparity (Nieder and Wagner 2001; Pettigrew and Konishi 1976a, b; Wagner and Frost 1993), and performs global shape analysis that goes beyond that performed by the primary visual cortex (V1; Nieder and Wagner 1999). The owl Wulst also displays clustered pinwheel arrangements of neurons sensitive to orientation, like the monkey and cat extrastriate cortex (Liu and Pettigrew 2003). Laterally eyed birds, such as pigeons, possess a much less differentiated Wulst lamination (Stacho et al. 2020), and no clustered orientation arrangements of pinwheels (Ng et al. 2010). The thalamofugal pathway in laterally eyed birds relates more to the processing of distant stimuli viewed in the monocular visual field (Budzynski et al. 2002; Budzynski and Bingman 2004) and spatial localization (Bischof et al. 2016; Watanabe et al. 2011).

## The tectofugal pathway

The tectofugal pathway plays the dominant role in detailed pattern vision in laterally eyed birds. This is particularly true when stimuli are viewed nearby in the frontal binocular visual field, as is mainly encountered in an operant chamber (Güntürkün and Hahmann 1999; Remy and Güntürkün 1991). The differentiated network of 15 layers comprising the avian optic tectum highlights the tectofugal pathways importance in both spatial attention (Marín et al. 2005) and stimulus perception (Neuenschwander et al. 1996; Neuenschwander and Varela 1993). The optic tectum displays a detailed retinotopic map of the visual field, and a progressive increase in the complexity of response properties and receptive field sizes at increasing depths (Frost and DiFranco 1976; Luksch 2003). Layer 13 of the optic tectum projects to the thalamic nucleus rotundus, by transforming the tectal retinotopy to a rotundal functionotopy for form, color, 2D motion and looming (Laverghetta and Shimizu 1999; Wang et al. 1993; Hellmann and Güntürkün 2001). These modules project topographically to the pallium that is composed of an inner region called the nidopallium, and a more dorsal region called the mesopallium. The nidopallium contains the main projection zone of the tectofugal pathway, which is known as the entopallium (Husband and Shimizu 1999) and also displays functional specializations for form/color and motion information along its anterior–posterior extent (Cook et al. 2013; Nguyen et al. 2004), and large receptive fields (Gu et al. 2002). The entopallium displays a topographic arrangement of cortex-like fiber connections oriented roughly perpendicular with the overlying intercalated nidopallium (NI), and mesopallium ventrolaterale (MVL) layers (Krützfeldt and Wild 2005; Stacho et al. 2020). These layers might be analogous with the mammalian extrastriate cortex (Butler et al. 2011; Karten 1969) and play a critical role in the categorization of complex visual stimuli.

In the following section, we will focus on the operation of the tectofugal projections in the telencephalon, as it is the best-understood cortex-like component of the visual system in birds in terms of its neurophysiology. These bottom-up visual computations form the basis of object, category, and abstract rule processing in birds, which in many tasks are executed at levels comparable to primates (Scarf et al. 2016; Veit and Nieder 2013).

## The avian visual cortex—perceptual categorization

Recent investigation of the physiology of the avian sensory cortex has revealed that hierarchical information processing builds increasingly complex and abstract representations of visual stimuli in the pigeon brain. These mainly feedforward shaped computations are very similar to the transformation of information observed across the mammalian ventral visual stream (Riesenhuber and Poggio 2000; Vinken et al. 2016). Arrays of neurons at higher stages of the processing hierarchy in mammals (such as primate inferior temporal cortex) are both selective to complex shapes and relatively invariant to non-linear changes, such as lighting, distance, viewpoint, and spatial translation (Bao et al. 2020; Freiwald and Tsao 2010; Gross and Schonen 1992; Wallis and Rolls 1997).

**Fig. 2:**
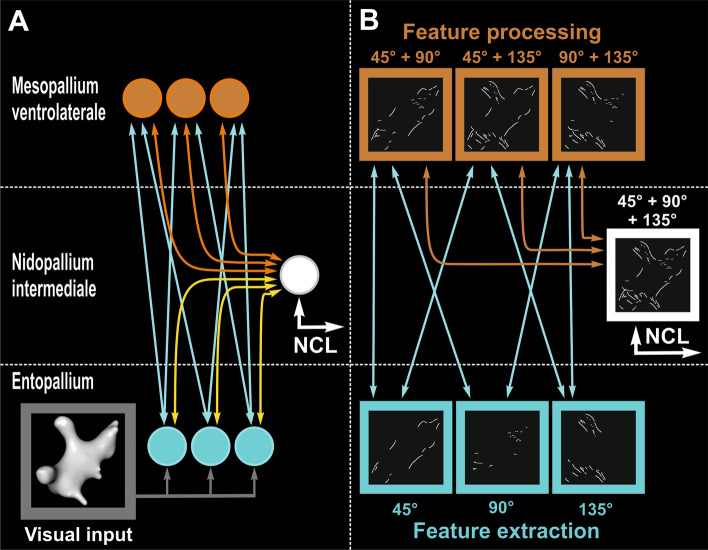
Hypothetical wiring pattern of the avian visual cortex and its proposed function on feature extraction and processing. **A** Hypothetical hierarchical visual information flow within the visual tectofugal pallium. **B** Depiction of the hypothesized hierarchical feature selection operations at different levels of the visual DVR. At the level of the entopallium, a basic feature selection operation is performed on the visual input (here depicted by a “digital embryo”, Pusch et al. 2022). In our example, this operation is represented by the detection of edges that roughly correspond to the depicted orientations. However, at the stage of the entopallium the processed visual information is not sufficient to signal information about an object category. At the next hierarchical level, information from several Entopallial neurons converges in MVL neurons and is integrated. The resulting population code at the level of MVL conveys information about an object category viewed by the pigeon. After a further computational step, the visual information of the MVL cells converges at the level of the NI and is transferred to higher associative areas

The entopallium is the first stage of hierarchical processing within the cortex-like architecture of the avian telencephalon that receives thalamic input and forwards information to the overlying MVL and NI layers to extract more complex features (Fig. 2A; Stacho et al. 2020; Clark and Colombo 2020). Consistent with entopallium reflecting a relatively early stage of categorization, neurons are selective for parameters such stimulus size and direction/speed of motion (Engelage and Bischof 1996; Gu et al. 2002), but the population responses do not distinguish well between images belonging to different stimulus categories (Fig. 2B; Azizi et al. 2019; Clark et al. 2022a). These features suggest that entopallium may reflect an intermediate stage of processing in the common elements model hierarchy of simple and complex unit layers (Serre et al. 2007; Soto and Wasserman 2012) that has not built sufficient receptive field invariance to discriminate between different object categories. Figure 2B illustrates these hypothetical feature selection operations within the visual cortex of pigeons.

Azizi et al. (2019) demonstrated that the population response of the overlying MVL layer distinguished between the features of images depicting animate and inanimate stimuli with far greater accuracy than at the level of the entopallium in a task that required the birds to peck the images for food reward without categorizing the stimuli. The visual features that the MVL population used to achieve categorization of the objects was also quite dissimilar from a simple V1-like model of Gabor filters, suggesting that MVL neurons represent more abstract features of stimuli than edges in particular orientations. Clark et al. (2022a) used a different image set and found that the population responses in MVL distinguished between the features of faces and scrambled faces with greater fidelity than the entopallium in a response-inhibition task that also did not require categorization (see Box 2 for further details). Interestingly, many MVL neurons respond strongly to scrambled images (Clark et al. 2022a, b) much like neurons in mammalian V1 (Vinken et al. 2016), suggesting that local edges are processed alongside some more abstract stimulus features at higher stages of processing within the cortex-like layers (cf. Fig. 2A, B). A preference for intact objects over scrambled images emerges at the level of NI (Clark et al. 2022b), suggesting that NI neurons sum the inputs of local orientation detectors at lower stages of processing to form receptive field filters that detect coarse low spatial frequency or complex shape features over a large area. The output of the NI layer is well situated to forward highly integrated visual information to the executive centers and memory systems of the avian brain (cf. Fig. 2A, B).

Box 2: Processing of categorical information in the different forebrain regions within the avian visual cortex—a machine learning approach A. Schematic representation of the visual neuronal components of the tectofugal pallial system and its constituent layers. The anatomical wiring between these layer-like entities is comparable to the canonical cortical microcircuit in mammals. The entopallium is the primary thalamic input zone to the avian telencephalon. Reciprocal connectivity between the entopallium and overlying mesopallium ventrolaterale (MVL) and intermediate nidopallial (NI) layers enables the generation of more sparse and complex coding of visual stimuli. The layer-like organization governing these abilities in birds may have converged on a similar solution to mammals to efficiently process the structure of visual objects. B. The neural coding principles of the avian visual system layers are similar to the hierarchical computations known to occur along the mammalian visual cortex. In two independent studies, a linear classifier was used to decode stimulus feature information from neuronal responses in the MVL. In this analysis, data points (i.e., average neuronal firing rates per stimulus presentation) were assigned to two different classes based on a linear category boundary. In a first step, the classifier is trained with correctly labeled data. Second, a non-labeled data set was analyzed by the trained classifier. The ability of the classifier to read out the category is then measured by the fraction of correctly classified data points. A growing number of MVL neurons accumulates categorical information and the categorization success incrementally increases as a function of the neuronal population size. In this example from Azizi et al. (2019), 33 MVL suffice to enable to neuronal population to discriminate the features associated with categories “animate” and inanimate” objects. A second study confirmed that the MVL population response conveys sufficient visual feature information to achieve accurate categorization of visual stimuli with highly distinct perceptual features (humans, scrambled humans, pigeons, scrambled pigeons, and sine wave gratings). The unfilled distributions (depicted in the white figure panels) show the performance of a linear discriminant analysis trained on randomly labeled firing rate data, which contained “no information” about the stimuli presented. The colored distributions show the performance of correctly labeled data. The classifier was run for 1200 iterations to generate a distribution of receiver operating characteristic (ROC) values. The y-axis depicts the number of samples, and the x-axis depicts the distribution of the receiver operating characteristic (ROC), a measure of the classification performance. The *p* values (and their error) are shown to the left for each group of stimuli viewed by the pigeons. The *p* value for each stimulus group is derived from how far away from the “no information” distribution of samples the correctly labeled performance falls. C. In contrast with MVL, no category information is readable from the entopallium population response. Despite an increasing cell population, the classification success for the category “animate” persists in the entopallium at low levels and does not distinguish between the features of different visual stimuli despite their very different physical appearance. These features are consistent with entopallium being situated at an early hierarchical stage of computation within the cortex-like layers. Results based on Azizi et al. (2019) and Clark et al. (2022a, b).
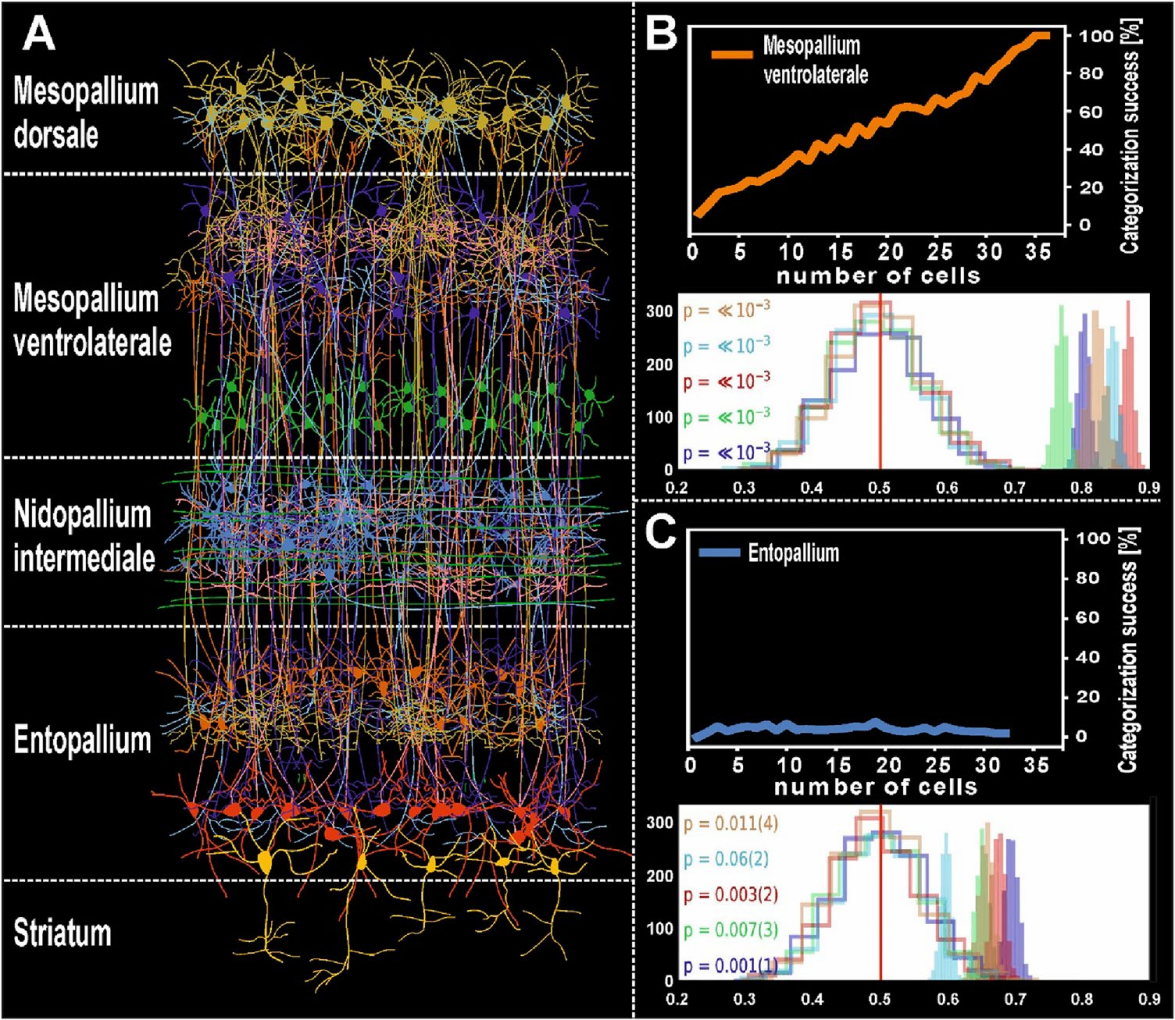

Both entopallium and MVL are also heavily involved in representing the distinction between rewarded and unrewarded stimuli, which is the first step towards forming category representations via the output of the cortex-like layers. Anderson et al. (2020) investigated the responses of entopallium and MVL neurons during a task requiring pigeons to categorize Monet and Picasso painting images. The responses of entopallium and MVL neurons both strongly distinguished between rewarded and unrewarded stimuli during the categorization task. Overall, the combined neurophysiological findings support the notion that preexisting physical similarity among stimuli is the main factor in the acquisition of visual categories in birds (Astley and Wasserman 1992), and indicates that reward-based feedback plays a key role in shaping the category membership of stimuli represented by the visual layers (Soto and Wasserman 2010). Recurrent feedback circuits between visual structures in the primate brain play a critical role in object identification and categorization by providing additional non-linear transformation operations on visual features than what a purely feedforward system can provide (Kar et al. 2019; Moeller et al. 2008). Investigation of the avian auditory system strongly suggests that categorisation, conceptualization, and rule formation in the avian brain also depends on top-down coding mechanisms that predict and map bottom-up sensory information to more abstract locations in perceptual space (Keller and Hahnloser 2009). Recurrent connectivity in the avian visual system is evident from neuroanatomy (Stacho et al. 2020) and is assumed an all relevant models discussed in the current review. However, a functional analysis of feedforward and feedback projections has not yet been performed at the physiological level. Determining how information propagates and cycles through the canonical circuits of the avian visual system to guide perceptual categorisation learning will be an important avenue of future research.

## The avian ‘prefrontal area’

A global analysis of the architecture of the avian forebrain revealed a network organization remarkably similar to the mammalian connectome (Fig. 1D; Shanahan et al. 2013). In both group of animals, distinct local networks are dedicated to different sensory modalities, motor, limbic, and executive processes. These local networks are connected through central hubs, one of which is the prefrontal cortex. In birds, this corresponds to the nidopallium caudolaterale (NCL). This executive hub takes on a central position with afferent and efferent projections to all associative, sensory limbic and premotor structures. While the NCL does not share the cortical columnar circuitry with the cortex (Stacho et al. 2020), several lines of evidence indicate that it is indeed the avian functional counterpart of the mammalian prefrontal cortex (Güntürkün et al. 2021). The NCL is usually identified as the part of the pallium with the richest dopaminergic innervation (Güntürkün 2005; von Eugen et al. 2020). A part of these dopaminergic terminals form ‘baskets’ as dense encapsulations of individual perikarya that enable a very specific targeting of individual neurons (Waldmann and Güntürkün 1993; Wynne and Güntürkün 1995). It is possible that this mode of innervation might have a similar functional role in the unlaminated cluster of the avian NCL as layer-specific projections in the mammalian PFC. At the functional level, the similarity to PFC was initially established with various lesion and inactivation studies that reliably demonstrated that NCL is involved in higher, more abstract processes such as the processing of behavioral rules (Güntürkün 1997a; Hartmann and Güntürkün 1998; Mogensen and Divac 1982; Diekamp et al. 2002a, b). These reports were confirmed in many neurophysiological studies that involved the NCL in many of the typical prefrontal functions (Güntürkün et al. 2021). To name a few examples, neural correlates of categorization (Kirsch et al. 2009; Ditz et al. 2022), working memory (Diekamp et al. 2002a, b; Hahn et al. 2021; Veit et al. 2014), executive control (Rose and Colombo 2005), reward processing (Koenen et al. 2013; Packheiser et al. 2021), numerosity (Wagener et al. 2018), rules (Veit and Nieder 2013), and even sensory consciousness as the ability to be aware of a sensory event (Nieder et al. 2020) have been discovered in NCL. Also, the neural ‘code’ found in the NCL largely follows the same principles as neural representations in the PFC. In working memory, neurons (‘delay cells’) show evidence of active maintenance (Diekamp et al. 2002a, b), capacity limitations can be accounted for by divisive normalization and neural oscillations are in line with modern bursting models of delay activity. In both the PFC and the NCL, the neurons are tuned in a highly flexible, task-specific way (Rigotti et al. 2013). This ‘mixed selectivity’ enhances robustness and flexibility as well as the ability to represent highly abstract information.

## The avian ‘prefrontal area’—perceptual categorization

We can think of categorization as a process that can occur at different levels of abstraction from physical stimulus properties (see Box 1 for formal definitions). The mammalian PFC and, correspondingly the avian NCL, are critical if abstraction increases. The location of the NCL within the avian pallial network allows the full integration of highly processed stimulus information from all modalities and the integration with limbic and, importantly, reward information. Unsurprisingly, neurons in PFC show categorical responses, that is they give a binary response even to a physically continuous stimulus. For instance, in a seminal experiment, Freedman et al. (2001) trained monkeys to categorize between renderings of cats and dogs. The stimulus set consisted of gradual morphs between cats and dogs, such that the stimuli were physically continuous. While neurons in inferior temporal cortex strongly responded to the physical ‘catness’ or ‘dogness’ of individual stimuli, prefrontal neurons gave a binary response as either cat or dog. In other words, prefrontal neurons did not represent the graded physical properties of the stimuli but only their category membership. The PFC is also able to flexibly respond to different categories. Interestingly, if the same stimulus set is categorized along different borders, then different groups of neurons represented the two categories (Roy et al. 2010). But if the animals flexibly switch between categorization involving different sets of stimuli, then the category representations were overlapping in the same neural population (Cromer et al. 2010). This highlights the importance of the PFC not only in rule-based categorization processes but also shows that conflicting, physically ambiguous categories require higher prefrontal involvement.

It is very likely that the category-selective response properties of NCL neurons are sculpted by reward and reward-driven dynamics of the strong dopaminergic input (von Eugen et al. 2020; Wynne and Güntürkün 1995) that activates local D1-receptors (Durstewitz et al. 1998). Their activation promotes synaptic stimulus–response associations (Herold et al. 2012) and signal the presence of predicted reward (Packheiser et al. 2021). In contrast, blocking of D1-receptors level the differential learning effects of unequal reward magnitudes (Rose et al. 2009, 2013; Diekamp et al. 2000). By the sum of these dopamine-mediated feedbacks, synaptic weights within cellular assemblies of NCL are increased and make it likely that the animal will increasingly select the rewarded stimulus category (Güntürkün et al. 2018; Soto and Wasserman 2010).

## The contributions of the asymmetric avian brain

Avian visual pathways reveal task-specific and complementary hemispheric asymmetries in chicken hatchlings, adult pigeons and many more avian species (Güntürkün et al. 2020a, b). In both chicks and pigeons, the left hemisphere excels in visual discrimination of various object features like patterns or color (Güntürkün 1985; Rogers et al. 2007; Skiba et al. 2002), while the right hemisphere is superior in object configuration (Yamazaki et al. 2007), social cognition (Deng and Rogers 2002a; Nagy et al. 2010; Rugani et al. 2015) and spatial attention (Chiandetti 2011; Diekamp et al. 2005; Letzner et al. 2017). These asymmetries pay dividends, since birds with pronounced behavioral asymmetries fare better in foraging tasks (Güntürkün et al. 2000; Rogers et al. 2004). When tested in the context of learning the category “human vs. non-human”, Yamazaki et al. (2007) demonstrated that both hemispheres approach this challenge with complementary contributions. While the left side of the brain exploited the diagnostic value of tiny visual features, the right hemisphere concentrated on the overall configuration of the sought category. Indeed, Manns et al. (2021) could show in an elegant study that both hemispheres can take the lead during categorization, possibly based on the perceptual strategy used.

When testing pigeons in conditioning chambers, they use their frontal visual field when categorizing stimuli. The stimuli are then perceived with the dorsotemporal retina which is mainly represented in the tectofugal system (cf. Fig. 1B; Güntürkün and Hahmann 1999; Remy and Güntürkün 1991) that has a bias for local processing of object features (Clark and Colombo 2022). In contrast, the thalamofugal pathway seems to participate in global processing of more distant objects in the surrounding of pigeons (Clark and Colombo 2022). Therefore, under ecological circumstances, both hemispheres likely complement each other during categorization when using the entire visual field. Since the neurobiological studies discussed below mostly derive from experiments conducted in conditioning chambers, they possibly primarily uncover the neural fundaments of a left-lateralized superiority of visual feature coding in the context of perceptual categorizations.

Structural and physiological asymmetries of the avian visual system were investigated in both chicken (Adret and Rogers 1989; Costalunga et al. 2022; Deng and Rogers 2002b; Rogers and Sink 1988) and pigeons (Güntürkün et al. 1998; Manns and Ströckens 2014; Ströckens et al. 2013). The emergence of such asymmetries require, at least in part, an asymmetrical epigenetic event during early development. Birds take an asymmetrical position in the egg such the left eye of the avian embryo is covered by its own body, while the right eye points to the eggshell. Every time the breeding adults stand up, light falls onto the eggs, traverses the eggshell and primarily stimulates the right eye (Buschmann et al. 2006). This is the starting point for the right eye/left hemispheric superiority in visual object discrimination in birds (Manns 2021). Obstructing visual input to the right eye by a patch before (Rogers and Sink 1988) or after hatch (Manns and Güntürkün 1999) reverses both behavioral and anatomical asymmetries. While chicken predominantly evince asymmetries in the thalamofugal pathway, pigeons mainly show asymmetries in the tectofugal system (Güntürkün et al. 2020a; b). In the following, we will focus on the situation in pigeons.

**Fig. 3:**
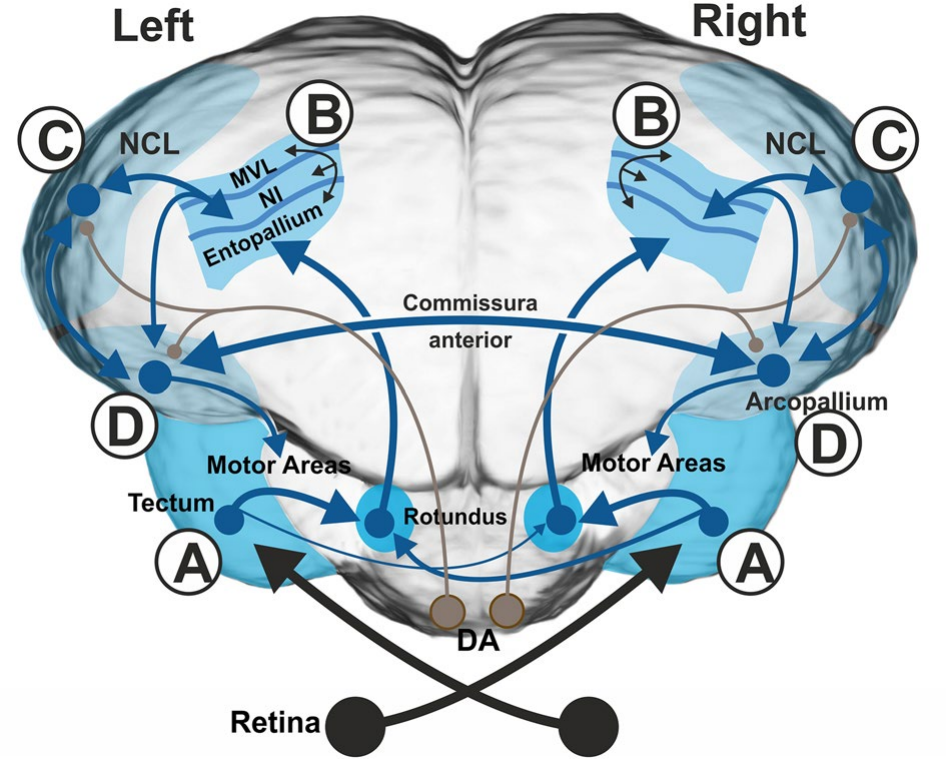
Anatomical depiction of the asymmetrically organized visual system of pigeons. Frontal view of the ascending tectofugal visual system along with the anterior commissure, the intrapallial projection of the tectofugal nidopallium intermediale (NI) onto the nidopallium caudolaterale (NCL) (**C**) and the projection of the NCL onto the motor arcopallium (**D**). The NCL receives input from the tectofugal system via the NI and feedback projections from the arcopallium (**C**). In addition, dopaminergic (DA) brainstem areas (shown in brown) modulate NCL and arcopallium activity patterns based on learning experiences. Note that the projection of the right tectum to the left rotundus entails more fibers (**A**), resulting in a more bilateral representation in the left rotundus and entopallium (**B**). Modified from Xiao and Güntürkün (2022)

Within the tectofugal pathway, already the first central structures show morphological and neurochemical asymmetries, indicating that bottom-up signals are processed in a lateralized manner (Güntürkün 1997b; Manns and Güntürkün 1999, 2003). In addition, contralaterally projecting tectal fibers are more numerous from the right tectum to the left rotundus than vice versa (Letzner et al. 2020; Fig. 3A, label A). Figure 3 summarizes the different asymmetrical processing steps and highlights their anatomical underpinnings using different labels (encircled letters A-D). These labels link the respective processing steps mentioned in the text and the figure.

This arrangement creates a more complete bilateral representation in the left rotundus that is then subsequently transferred to the left entopallium (Fig. 3, label B; Güntürkün et al. 1998; Letzner et al. 2020). This tectofugal asymmetry of visual representation could meanwhile be verified with behavioral (Güntürkün and Hahmann 1999; Valencia-Alfonso et al. 2009) as well as electrophysiological techniques at thalamic (Folta et al. 2004, 2007) and telencephalic levels (Verhaal et al. 2012; Xiao and Güntürkün 2022). However, it is important to keep in mind that the tectofugal visual system is not only a feedforward pathway but also includes feedback loops. For example, rotundal neurons also receive top-down pallial information that is relayed via the optic tectum. Folta et al. (2004) and Freund et al. (2016) could reveal that left rotundal neurons were strongly modulated by top-down input from the visual Wulst, while those in the right rotundus were hardly modified by descending signals. This implies that mainly left-sided thalamic neurons receive feedback from higher visual areas such as the Wulst. This finding has two implications: first, it shows that thalamo- and tectofugal pathways are not only parallel but also highly interconnected systems—an aspect that is often overlooked. Second, such a top-down asymmetry could modify left hemispheric thalamic neurons by experience-based telencephalic input, in order to selectively increase the activity level of those thalamic neurons that process category-relevant visual stimuli. Indeed, lateralized cortical top-down signals in human subjects modify activity patterns of downstream areas during categorization (Coutanche and Thompson-Schill 2015). This asymmetry of top-down control is altered by learning diagnostic stimulus features which then are pre-activated in lower sensory areas (Sigala and Logothetis 2002; Ullman 2007). At the cellular level, the results in pigeons reveal that similar processes could also occur already at thalamic level and may modify hemispheric left–right differences of stimulus categorization.

## Avian categorization at neural level—a mechanistic summary

**Fig. 4:**
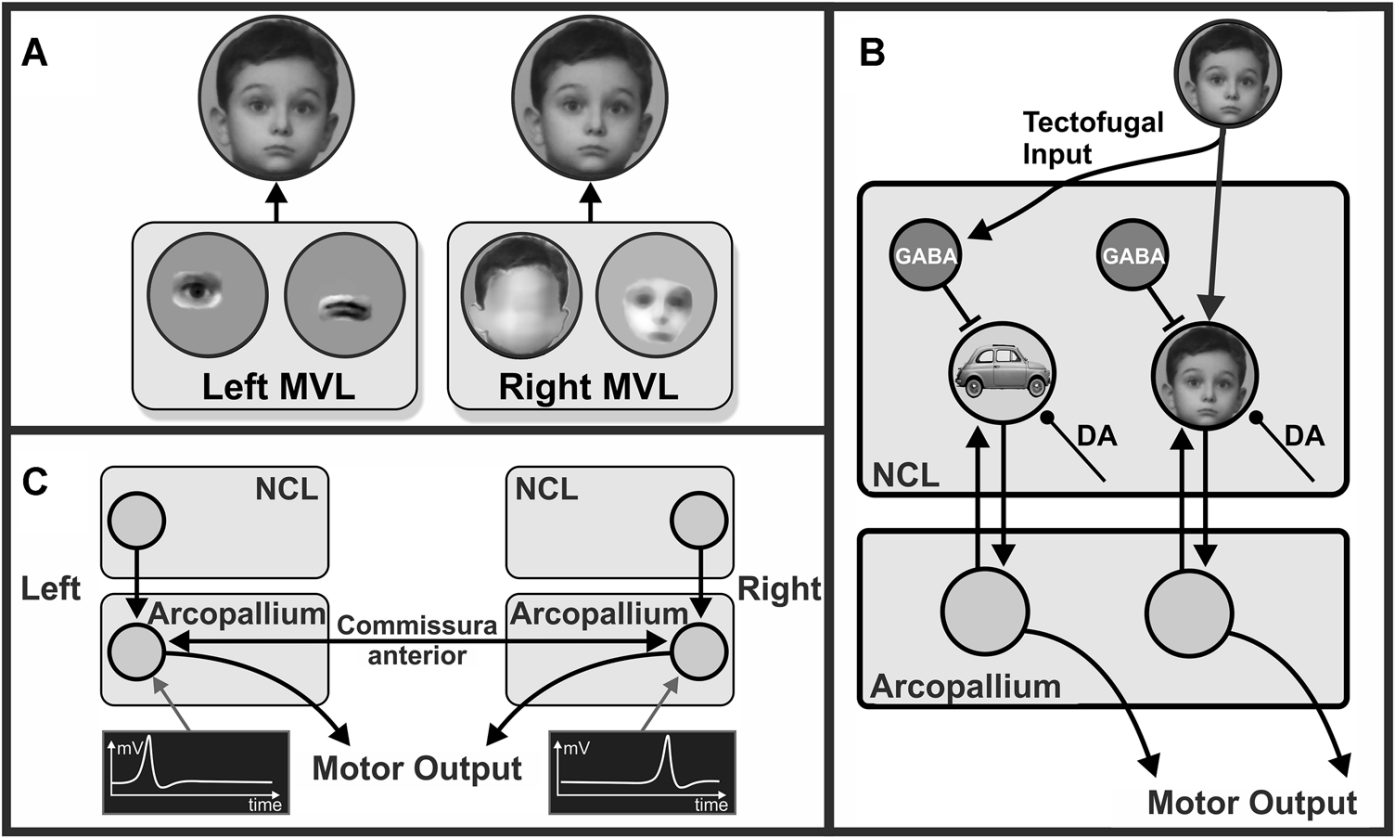
Hypothetical depiction on how visual perceptual categorization is realized in the asymmetrically organized visual system of pigeons. The categorization of humans (S+) vs. cars (S−) is used as an example. **A** Depiction of two neurons in the left and two in the right mesopallium ventrolaterale (MVL). Left MVL neurons respond to small diagnostic facial features while right cells are activated by stimulus configurations that indicate the presence of a human face. **B** The tectofugal input inhibits “car”-coding NCL neurons via inhibitory interneurons and activates cells that code for the category “human”. Dopaminergic input signals the presence or absence of reward and can thus increase synaptic weights between assemblies consisting of tectofugal, NCL, and arcopallium neurons that are all activated when the pigeon pecks onto a stimulus that belongs to the category “human”. Circuitry according to Ditz et al. (2022). **C** The commissura anterior connects the arcopallia of both hemispheres. When, based on diagnostic features, the left hemisphere detects a stimulus of the “human”-category, it can delay the latency of the action potentials of competing right arcopallial cells, such that their motor output is activated too late to control the choice of the animal

Based on these results, we will now outline a hypothesis on the neuronal processes during visual feature discrimination in birds (Fig. 4). This hypothesis also incorporates a proposal on how the differently specialized hemispheres could switch between modes of interhemispheric competition and hemispheric cooperation.

As outlined above, both hemispheres make different contributions for the visual analysis of various stimuli. If a bird has to categorize, say, humans from cars, left MVL cells will very likely exploit small diagnostic facial details like eyes, nose, and mouth to categorize pictures of humans at the population level (Azizi et al. 2019; Koenen et al. 2016). In contrast, right MVL neurons will mainly respond to the configurations of the body and the face of humans (Fig. 4A).

Ditz et al (2022) developed a data-driven model on the dynamics of NCL microcircuits of crows that worked on a demanding numerical categorization task. According to their results, the appearance of a stimulus (say, a human) would rapidly activate putative inhibitory interneurons that are broadly tuned to other categories than “human” and thus can exert a widespread and fast inhibitory feedforward effect on a large number of diversely tuned NCL projection neurons. As a result, network activity to, say, cars and other objects are dampened. After a short delay, putative projection neurons are activated that respond to the appearance of a human stimulus. In contrast to interneurons, these cells are narrowly tuned and only selectively respond to the sought stimulus class, while inhibitory interneurons in the vicinity of “human”-coding NCL neurons are inhibited. By such an arrangement, only cells that respond to the correct category remain active and control the response of the animal (Ditz et al. 2022) (Fig. 4B). The results of Ditz et al. (2022) in crows nicely overlap with those from pigeons. Like in crows, single unit recording studies in pigeon NCL and (pre)motor arcopallium reveal, that the inhibitory effect of the non-rewarded stimulus is faster and less precisely tuned than the excitatory effect of the rewarded one (Xiao and Güntürkün 2018; 2022). Thus, network dynamics are similar in crows and pigeons.

Data from pigeons outline how asymmetries of categorization are constituted. Both in NCL and in arcopallium, the speed of stimulus encoding during stimulus discriminations did not differ between left and right hemispheres. In contrast, the cellular timing of action generation was faster in the left hemisphere since the majority of left hemispheric neurons reached their maximal spiking frequency just before response execution, while those of right hemispheric cells were slow and came too late to control the response of the animal. Thus, left hemispheric neurons dominated the birds’ behavior not by a higher categorization ability, but by their speed in monopolizing the execution of the decision. This critical left–right difference was realized by differences of left–right interactions via the commissura anterior that connects the arcopallia of both hemispheres (Letzner et al. 2016). Xiao and Güntürkün (2018) showed that the left arcopallium delayed the peak activity time of contralateral right arcopallial neurons (Fig. 4C). As a result, the output of right hemisphere cells often came too late to control the choice of the animal. Thus, interhemispheric interactions in birds do not simply activate or inhibit the other hemisphere, but accelerate or decelerate cellular response speed in the other hemisphere, thereby establishing unilateral control on the animals’ decision (Xiao and Güntürkün 2018).

## From categories to concepts

It makes sense to distinguish categories from concepts, although this distinction is not sharp but of a transitional nature. In our view, a category is defined by overlapping perceptual features. These constitute the core of the common elements theory when applied to categories. In contrast, concepts are constituted by groups of stimuli that do not all share these perceptual features. Still, humans and some other animals might conceive them as a common group.

**Fig. 5:**
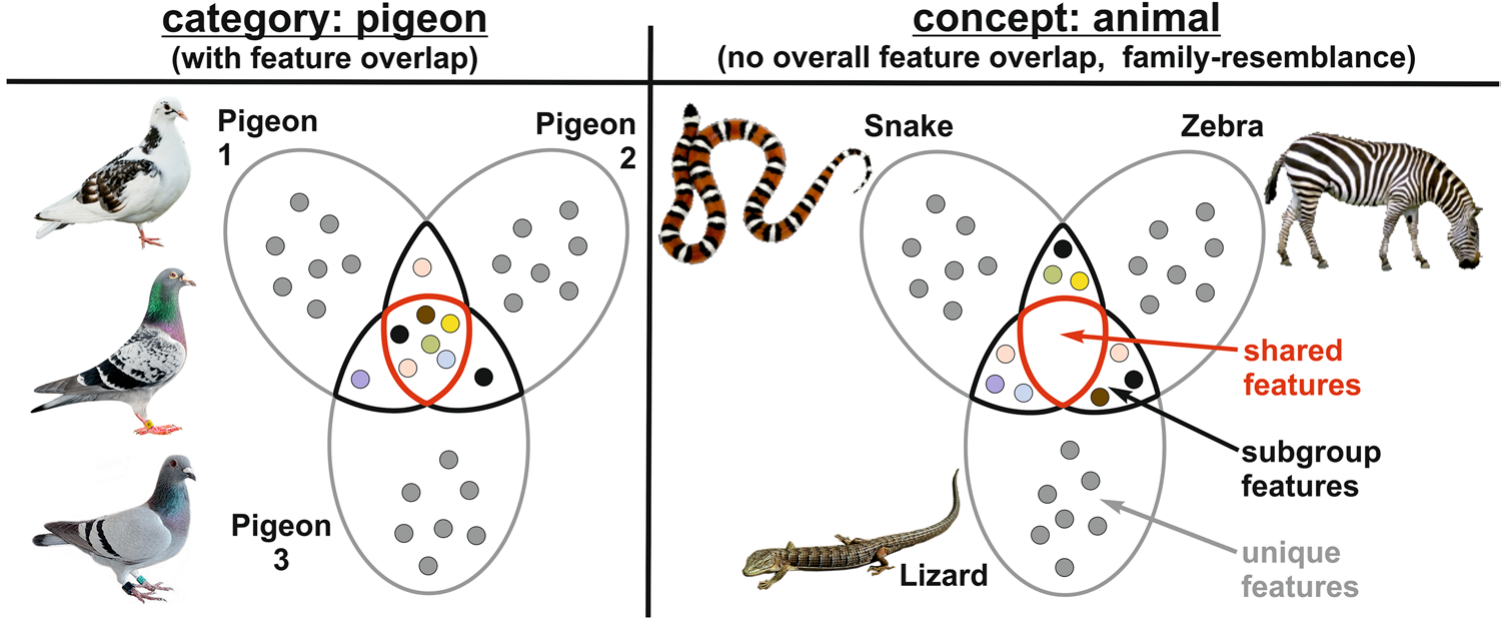
Schematic depiction of a hypothesis on how categories and concepts emerge. The left panel exemplifies the category “pigeon”. Each individual category member is characterized by a set of idiosyncratic features. These unique stimulus elements render the different pigeons identifiable. Further, some individuals might share visual aspects leading to subgroup features. However, the defining component of the category “pigeon” are overall shared features—visual aspects that are common to all category members. These shared features lead to a robust representation of a category based on visual similarity. The right panel depicts the situation for the concept “animal”, a concept that pigeons learn with some additional training (Roberts and Mazmanian 1988). As for categories, unique features characterize each individual instance of the concept. Further, several instances might have visual subgroup features. However, no features are shared by all instances of the category. As a result, no combined representation based on shared visual similarity is possible, but family resemblance emerges based on the presence of multiple subgroup features across all stimuli

The emergence of categories and concepts has been recently investigated in a modeling-study using a deep neural network (Henningsen-Schomers and Pulvermüller 2021). Here, visual features that are present in all stimuli of the sought category (e.g., shared visual features of pigeon breeds, Fig. 5 left) create common elements of this category (overlapping dots shared by all stimuli). In addition, some elements are only shared by a subgroup of stimuli. The situation is different for abstract concepts. Their features were never shared across all members belonging to this concept, but only between subgroups of stimuli. Thus, as visible in Fig. 5 (right panel), the central zone of the concept is empty, while the overlapping zones between neighboring stimuli contain shared elements. This arrangement results in an intermediate state of feature overlap called family resemblance.

After training the network with instances of such category members, the emerged cell assemblies were investigated. As a result, the authors found that stimuli belonging to a perceptual category (left side of Fig. 5) were represented in cell assemblies that showed category defining features in the neural network’s central connector hub area. This result is due to the effect that units coding for shared features are activated most frequently, leading to a relative suppression of the neurons responsive for unique features. If the common core is sufficiently activated, the categorical cell assembly will ignite as a whole, resulting in a strong persistence throughout task execution. In parallel, representation of unique features or subgroup features that are shared by only a few members of a category might pale, which results in an overshadowing of these features.

This is different for concepts. Here, no joint and shared features exist. In contrast, a larger number of neurons code for elements that are shared by only subgroups of the concept. Exactly the sum of all of these subgroup features could represented concepts. The stronger reliance on subgroup feature neurons in case of concepts creates the “family resemblance” and contextual dependency. Indeed, in humans abstract concepts do rely much more on its contextual embedding than perceptual categories (Schwanenflugel et al. 1988; 1992).

These modeling results (Henningsen-Schomers and Pulvermüller 2021) fit well with behavioral data showing that perceptual categories that share many stimulus details are easier to learn and categorize than more abstract categories at the superordinate level (see Box 1 for formal definitions; e.g., Lazareva 2004). Further, pigeons have severe problems mastering choice tasks using polymorphous concepts, i.e., stimuli that are defined as category members if they contain m-out-of-n stimulus features (Lea et al. 2006; von Fersen and Lea 1990). One explanation of this behavioral finding might be that these concepts need more behavioral training due to their neurocomputational demands to learn a group structure that lacks a central connector hub with common elements (Henningsen-Schomers and Pulvermüller 2021). If learning a concept requires the acquisition of a large number of context-dependent subgroups of features that jointly create a concept, it is easy to see, that animals with more pallial neurons can be ranked according to the speed of learning of a concept (Wright et al. 2003; Güntürkün et al. 2017). This might also explain why crows with their much larger number of associative pallial neurons are able to master these kind of tasks with ease, while pigeons face a hard time (Veit and Nieder 2013; Ströckens et al. 2022). In conclusion, sufficient training and computational power in associative brain structure might enable abstract concepts to evolve in various animal species.

## The forthcoming frontiers

The synopsis of recent findings from anatomical studies, behavioral experiments, electrophysiological recordings and modeling attempts allow the formulation of a coherent theory of perceptual categorization and concept formation. Now, these theoretical implications need to be experimentally verified. In parallel, several methodological aspects might be worth to consider in future experiments on perceptual categorization and concept formation.

At the behavioral level, several algorithms to generate stimuli were introduced, which are geared to probe critical features used by the animals to facilitate categorization (Apostel and Rose 2021; Hegdé et al. 2008; Pusch et al. 2022). These stimuli represent artificial yet naturalistic objects that are free of human semantics but based on features for class distinction that can be tracked by the experimenter. Taking this approach a step further, genetic algorithms are used to adaptively change stimulus features during one experimental session, for instance to find optimal stimulus parameters for the animals (Qadri and Cook 2021). Such stimuli also allow a near perfect control over the statistics of the stimuli that define a category and might help to uncover the aspects, elements and features that guide the choice behavior of the animals.

The level of analysis might also benefit from the inclusion of additional behavioral parameters. One approach used in recent experiments is peck-tracking. Similar to human eye tracking, the peck location of the pigeons signaling their choice can be used as a proxy for measuring the pigeon’s visual attention. Indeed, it has been shown that pigeons, when learning to categorize visual stimuli, allocate their attention to the predictive features of the stimuli reflected by an increased pecking rate onto these stimulus aspects (Castro et al. 2021; Castro and Wasserman 2017; Dittrich et al. 2010; Pusch et al. 2022). In combination with the aforementioned stimulus material, this information might further aid the understanding of which stimulus features gain control over the elicited behavior.

This principle can be extended far beyond peck-tracking. Modern video analysis, such markerless pose estimation, allows tracking of behavioral aspects that were previously difficult to systematically incorporate in a detailed analysis (for example using DeepLab Cut: Nath et al. 2019; Wittek et al. 2022). All these approaches reduce experimenter biases and can reveal details not obviously visible in aggregated data to achieve an ecological valid and unbiased behavioral analysis (Anderson and Perona 2014).

On the neurophysiological level, the analysis of the supposed neural computations within the sensory aspects of the dorsal ventricular ridge (DVR)—a large pallial collection of nuclei that bulge below the lateral ventricle—and their connections with the NCL constitute core future questions. But these questions extend beyond the areas that were covered in this review and should incorporate key areas such as striatum and hippocampus. Both structures very likely constitute key contributors to categorization learning. Recent approaches like visual discrimination learning in awake and actively working pigeons tested with in ultrahigh magnetic field imaging systems, could aid these analyses, by visualizing with high resolution all cerebral areas that participate in certain task components (Behroozi et al. 2020). This further highlights the fact that categorization—like all cognition—cannot be understood at the level of individual neural structures but it must be seen as a network-process. The use of high-density methods such as electrophysiological recordings with silicone-probes, can allow parallel data-collection from the entire stacked avian visual cortex or even bilaterally from the visual and prefrontal structures simultaneously. The data that is generated with these approaches allows for the analysis of the temporal dynamics and population-level processes within and between the different nodes of the network. These critical tests might allow to further discern the network-level processes that underlie categorization and concept formation. Methods such as optogenetic stimulation and inhibition (Deisseroth 2011; Rook et al. 2021) further complement this approach by allowing causal interventions targeting for example top-down processes in perceptual categorization.

Last but not least, the differences in concept learning between pigeons and crows exemplifies important species differences within the avian class. These differences should be turned into important heuristic opportunities that enables us to see how ecological embedding and neural specialization affect the different components of avian cognition. This is only possible with a larger number of avian species that are tested.

Taken together, theoretical implications as well as methodical and conceptual advancements provide the opportunity for future experiments that will broaden our understanding of perceptual categorization in birds.

## Data Availability

This review contains no novel data but provides an overlook of results reported in publications. Please refer to the data availability statements in these original papers.
